# The expression and prognostic value of disulfidptosis progress in lung adenocarcinoma

**DOI:** 10.18632/aging.204938

**Published:** 2023-08-07

**Authors:** Lina Ni, Huizhen Yang, Xiaoyu Wu, Kejin Zhou, Sheng Wang

**Affiliations:** 1 Department of Respiratory, Jinhua Guangfu Cancer Hospital, Jinhua, Zhejiang 321200, China

**Keywords:** lung adenocarcinoma, disulfidptosis, prediction model, scRNA-seq, prognosis

## Abstract

Disulfidptosis is a new cell death model caused by accumulating intracellular disulfides bonding to actin cytoskeleton proteins. This study aimed to investigate the expression and prognostic value of disulfidptosis-related genes (DRGs) in lung adenocarcinoma (LUAD). The data of expression profiles and scRNA-seq were collected from TCGA and GEO databases. The different expressions of DRGs between normal and LUAD tissues were compared. The LASSO analysis and multivariate Cox regression analysis were utilized to develop a DRGs model for the prognosis evaluation in LUAD. The model’s predictive accuracy was evaluated with the area under the receiver operating characteristic curve (AUC) and C-index. Survival analysis, univariate and multivariate Cox regression analysis were used to assessing the predictive value of the DRGs model. ScRNA-seq data were analyzed with “Seurat” and “Monocle 2” packages. There were significant differences in 22 DRGs between normal and tumor tissues. A model with five DRGs (ACTB, FLNB, NCKAP1, SLC3A2, SLC7A11) was constructed. The AUC and C-index of the model were significantly higher than that based on clinical parameters. Survival analysis, univariate and multivariate Cox regression analysis demonstrated risk score was an independent prognostic predictor. In the scRNA-seq study, we identified 14 clusters and 11 cell types. Clusters 2, 8, and 13 were annotated into Epithelial cells. SLC7A11 and SLC3A2, NCKAP1 and FLNB, ACTB expressed most abundantly in Epithelial cells, Endothelial cells, Naive CD4 T, respectively. We explored the expression of DRGs in LUAD and constructed a predictive DRGs model, which was stable and reliable for predicting LUAD prognosis.

## INTRODUCTION

It was reported that lung cancer (LC) was one of the most common malignant tumors worldwide and the leading cause of cancer-related mortality [1]. In 2020, over 2.2 million new cases were diagnosed as LC globally, and more than 1.8 million people died of LC. As the primary subtype of LC, lung adenocarcinoma (LUAD) accounts for more than 40% of LC cases [2, 3]. In recent years, new diagnostic techniques and treatment strategies, such as immunotherapy, have been emerging and prominently prolonged the survival time of LUAD patients [3]. However, the 5-year survival rate of LUAD patients remains <20% [4, 5]. Therefore, there is an urgent need to identify more biomarkers for diagnosis and prognostic assessment in LUAD, which may be helpful to risk stratification and increase the long-term survival rate of LUAD patients.

Programmed cell death, such as apoptosis and autophagy, could enable cells to coordinate their end, thereby benefiting living organisms [6, 7]. Recently, Liu et al. proposed a novel form of cell death named disulfidptosis. The main mechanism of disulfidptosis is that accumulating intracellular disulfides caused by cells starved of glucose in SLC7A11^high^ cells could bond in actin cytoskeleton proteins [8, 9]. In addition, excessive cystine uptake could couple with the insufficient supply of NADPH, causing NADPH depletion, aberrant disulfide bonding in actin cytoskeleton proteins, F-actin collapse, and subsequent cell death [8]. Distinct from apoptosis and ferroptosis, deleting BAX and BAK, fatty acid oxidation inhibitors, did not prevent death, and iron was not required [8]. The results reported a new cell death model, disulfide stress-mediated disulfidptosis, and a viable therapeutic strategy to target disulfidptosis in cancer treatment. However, the disulfidptosis process in LUAD and relevant disulfidptosis-related genes related to the prognosis remains to be further elucidated.

This study collected mRNA expression profiles from The Cancer Genome Atlas (TCGA; https://www.cancer.gov) and Gene Expression Omnibus (GEO; https://www.ncbi.nlm.nih.gov) to investigate the expression of disulfidptosis-related genes (DRGs). Then, we utilized disulfidptosis-related genes to construct a DRGs model for predicting the clinical outcome in LUAD. Lastly, we explored the expression profiles of disulfidptosis-related genes with Single-cell RNA sequencing (scRNA-seq).

## RESULTS

### The expression profile of 23 DRGs

**Figure 1:**
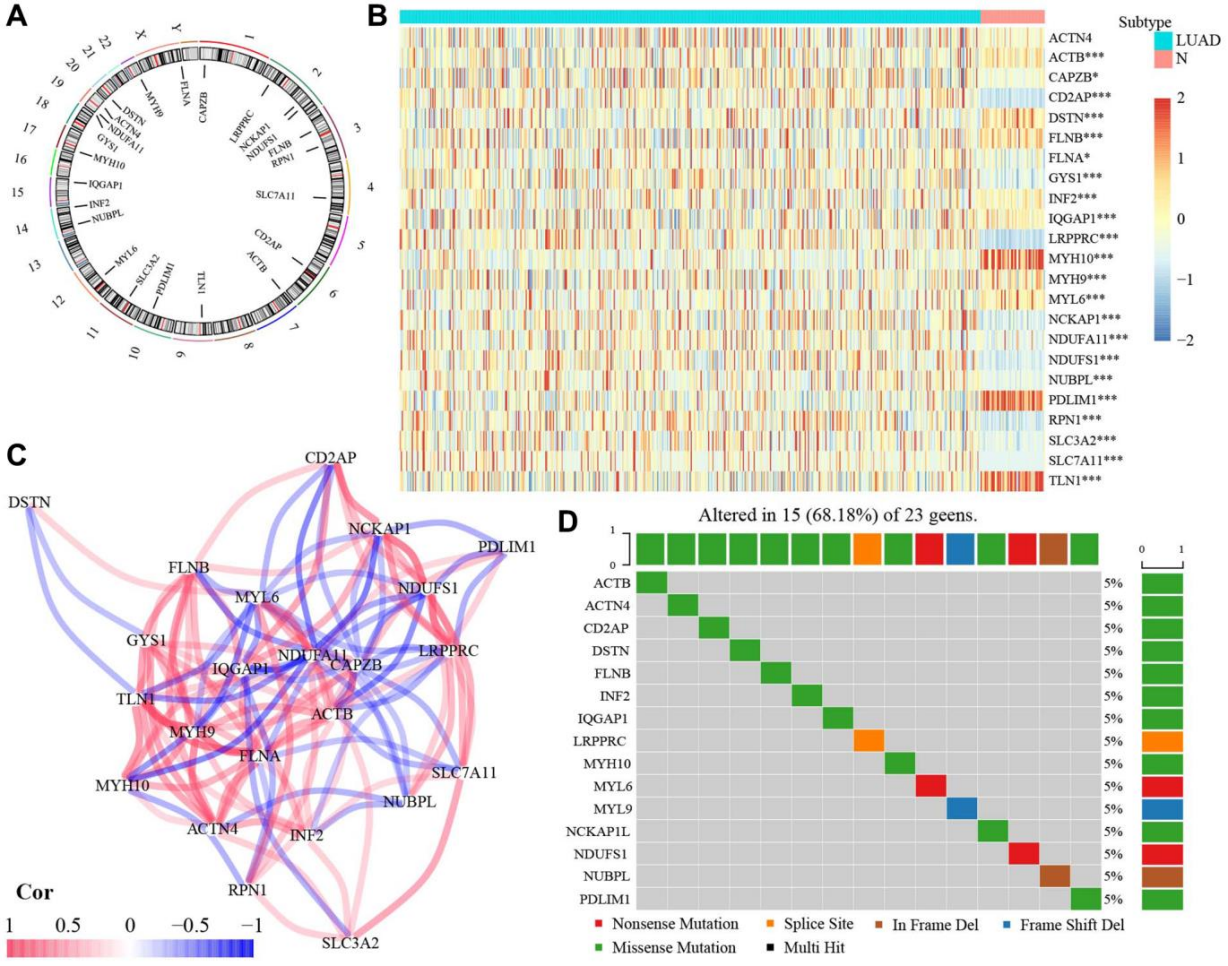
**The expression of 23 DRGs in LUAD.** (**A**) Location of 23 DRGs in chromosomes. (**B**) The different expression of DRGs between normal and LUAD tissues. (**C**) The correlation network of 23 DRGs. (**D**) The mutation frequency of 23 DRGs. Abbreviations: DRGs: Disulfidptosis-related genes; LUAD: Lung adenocarcinoma; N: normal tissues. ^*^P < 0.05, ^**^P < 0.01, ^***^P < 0.001.

As shown in Figure 1A, among 23 DRGs, 3 DRGs (GYS1, NDUFA11, ACTN4) were located at Chromosome 20. In addition, 3 DRGs (LRPPRC, NCKAP1, NDUFS1) were situated at Chromosome 2. 2 DRGs (FLNB, RPN1) were found at Chromosome 3. 2 DRGs (NUBPL, INF2) were located at Chromosome 14. Others (CAPZB, SLC7A11, CD2AP, ACTB, TLN1, PDLIM1, SLC3A2, MYL6, IQGAP1, MYH10, DSTN, MYH9, FLNA) were located at Chromosome 4, 6, 7, 9, 10, 11, 12, 15, 17, 21, 22, and X, respectively. The Wilcoxon rank-sum test showed significantly different expressions in 22 DRGs, except for ACTN4, between normal and tumor tissues (Figure 1B). The correlation among 23 DRGs was presented in Figure 1C. Among 23 DRGs, 15 DRGs had gene mutation, although the mutation frequency for each DRGs was relatively low (Figure 1D).

### Establishment and evaluation of a DRGs model

**Figure 2:**
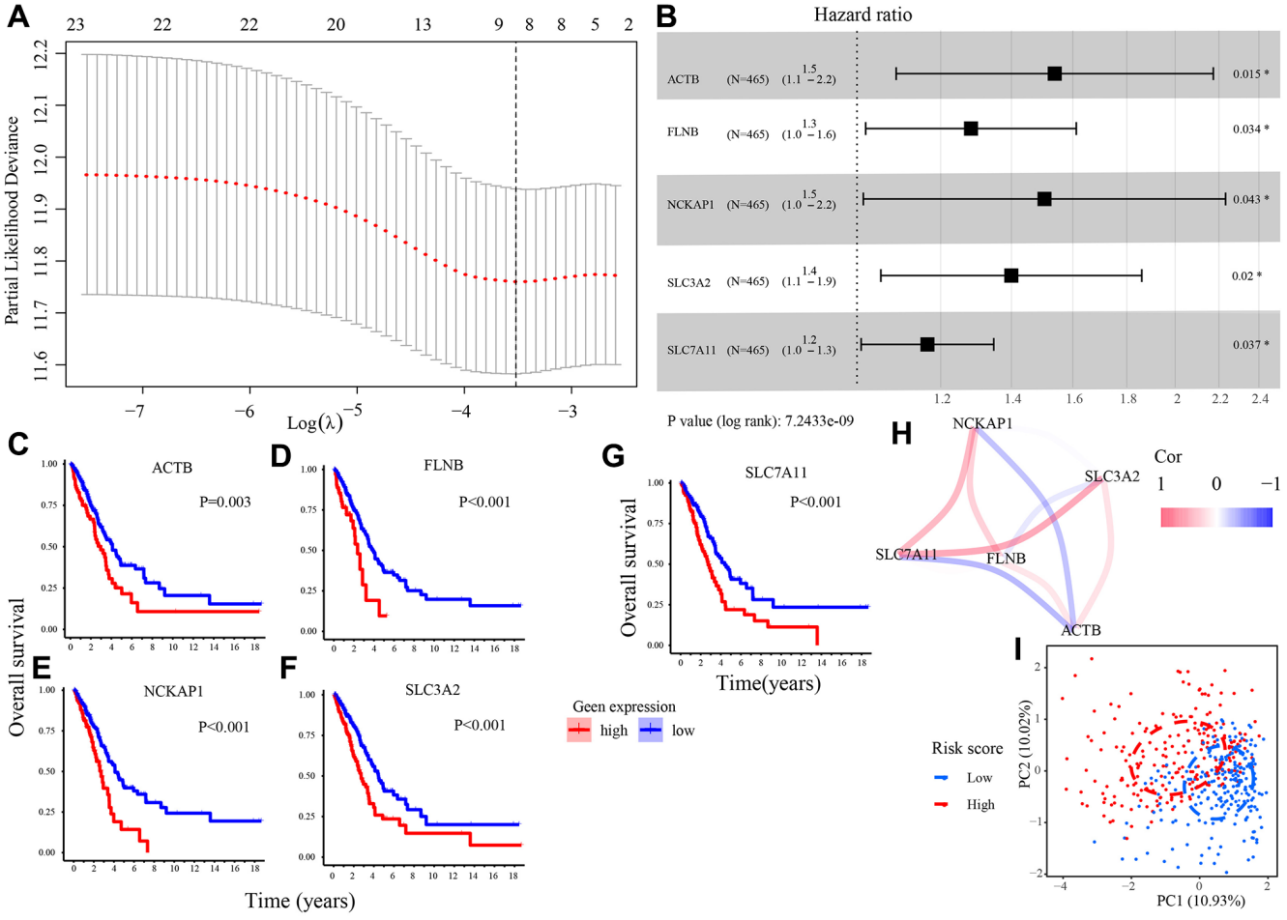
**Construction of a DRGs model in TCGA set.** (**A**) The LASSO regression analysis to filter out prognosis-related DRGs. (**B**) The multivariate Cox regression analysis to develop a DRGs model. (**C**–**G**) Survival analysis of 5 candidate DRGs (ACTB, FLNB, NCKAP1, SLC3A2, SLC7A11). (**H**) The correlation network of 5 candidate DRGs. (**I**) Patients in different risk groups gathering in two areas in PCA analysis. Abbreviations: LASSO: Least absolute shrinkage and selection operator; PCA: Principal component analysis.

We utilized the least absolute shrinkage and selection operator (LASSO) regression (LASSO) analysis to analyze 23 DRGs and determined 8 DRGs highly related to the overall survival (OS) of LUAD patients in the TCGA set (Figure 2A). Then, those 8 DRGs were subjected to the multivariate Cox regression analysis. 5 candidate DRGs (ACTB, FLNB, NCKAP1, SLC3A2, SLC7A11) were screened out and used to build a prognostic model (Figure 2B) and a risk score formula to calculate the risk score of all patients. The risk score formula was presented as follows. The Kaplan–Meier plots demonstrated that all 5 DRGs were unfavorable factors for the clinical outcomes of LUAD patients (Figure 2C–2G). Figure 2H showed the correlation among 5 DRGs. According to the optimal cut-off risk score: 0.987, patients were divided into low- and high-risk groups. The principal component analysis (PCA) revealed that the samples in two risk groups were distributed in different areas (Figure 2I).


$$\begin{array}{l} \text{Risk score}=(0.430\;\times \;\exp\text{of ACTB})\;\\ \;\;\;\;\;+\;(0.248\;\times \;\exp\text{of FLNB})\;\\ \;\;\;\;\;+\;(0.408\;\times \;\exp\;\text{of}\;\text{NCKAP1})\\ \;\;\;\;\;+(0.336\;\times \;\exp\;\text{of}\;\text{SLC3A11})\\ \;\;\;\;\;+\;(0.153\;\times \;\exp\;\text{of}\;\text{SLC7A11}) \end{array}$$

**Figure 3:**
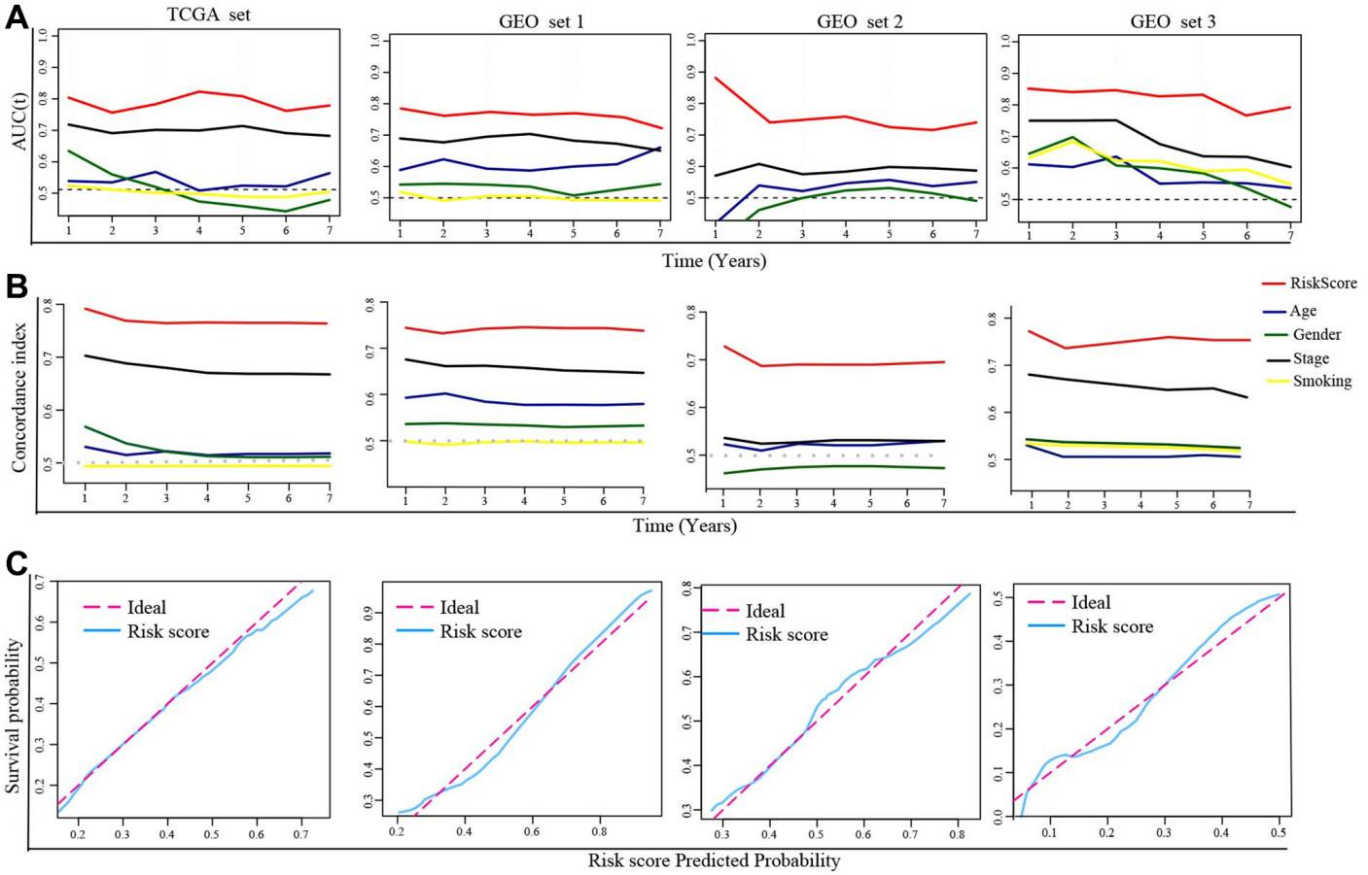
**Evaluation of the DRGs model.** (**A**) The time-dependent AUC value in each set. (**B**) The time-dependent C-index in each set. (**C**) The calibration plots of each set. Abbreviations: AUC: The area under the curve; C-index: concordance index.

Next, we calculated the area under the curve (AUC) value and the c-index in each set to estimate the predictive accuracy of the DRGs model. The results showed the AUC values and c-index based on risk score in all sets were higher than that based on clinical indexes (age, gender, smoking, TNM stage), hinting that the DRGs model performed well in predicting clinical outcomes of LUAD patients (Figure 3A, 3B). In addition, the calibration plot in each set revealed that when the line angle is 45°, it represents the best prediction result, indicating that the DRGs model had a good predictive ability (Figure 3C).

### Correlation between DRGs model and clinical characteristics

**Figure 4:**
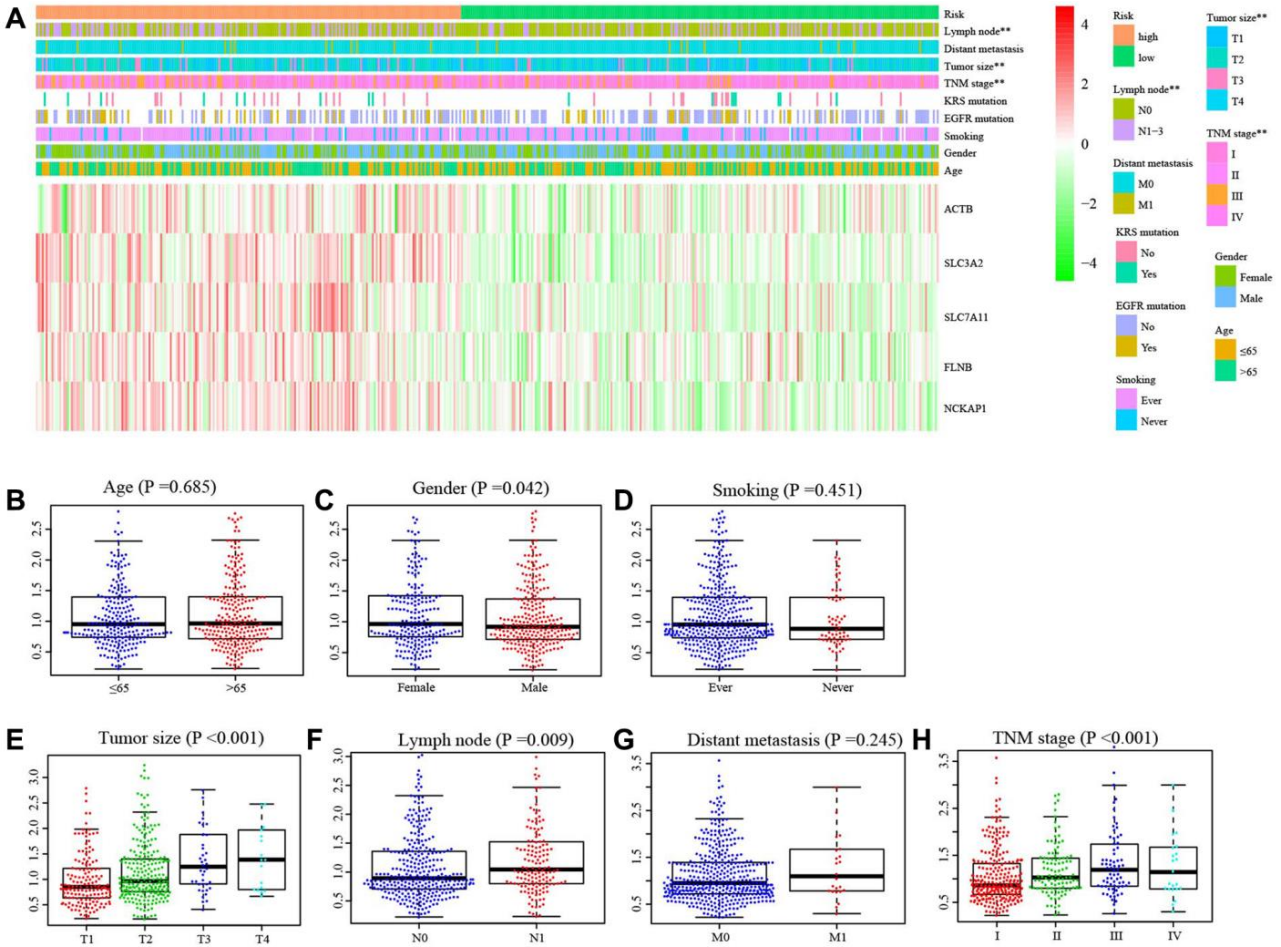
**Correlation between risk score and clinical characteristics.** (**A**) The distribution of clinical characteristics between high- and low-risk groups. (**B**) The difference of risk score between Age ≤65 and Age >65, (**C**) different gender, (**D**) smoking and non-smoking, (**E**) tumor size, (**F**) with and without lymph node metastasis, (**G**) with and without distance metastasis, (**H**) different TNM stage. ^**^P < 0.01.

The distributions of samples with different TNM stage (*P* = 0.003), tumor size (*P* < 0.003), and lymph node metastasis (*P* = 0.025) were significantly diverse between different risk groups (Figure 4A). Moreover, there was a significant difference in the risk scores among patients with varying tumor sizes (*P* < 0.001, Figure 4E). Similar results were observed among patients with different TNM stages (Figure 4H). The risk score significantly increased in female patients (*P* = 0.042, Figure 4C) or with lymph node metastasis (*P* = 0.009, Figure 4F). However, we did not find the effect of age (Figure 4B), smoking (Figure 4D) and distant metastasis (Figure 4G) on the risk score.

### The DRGs model is an independent prognostic factor for the prognosis of LUAD

**Figure 5:**
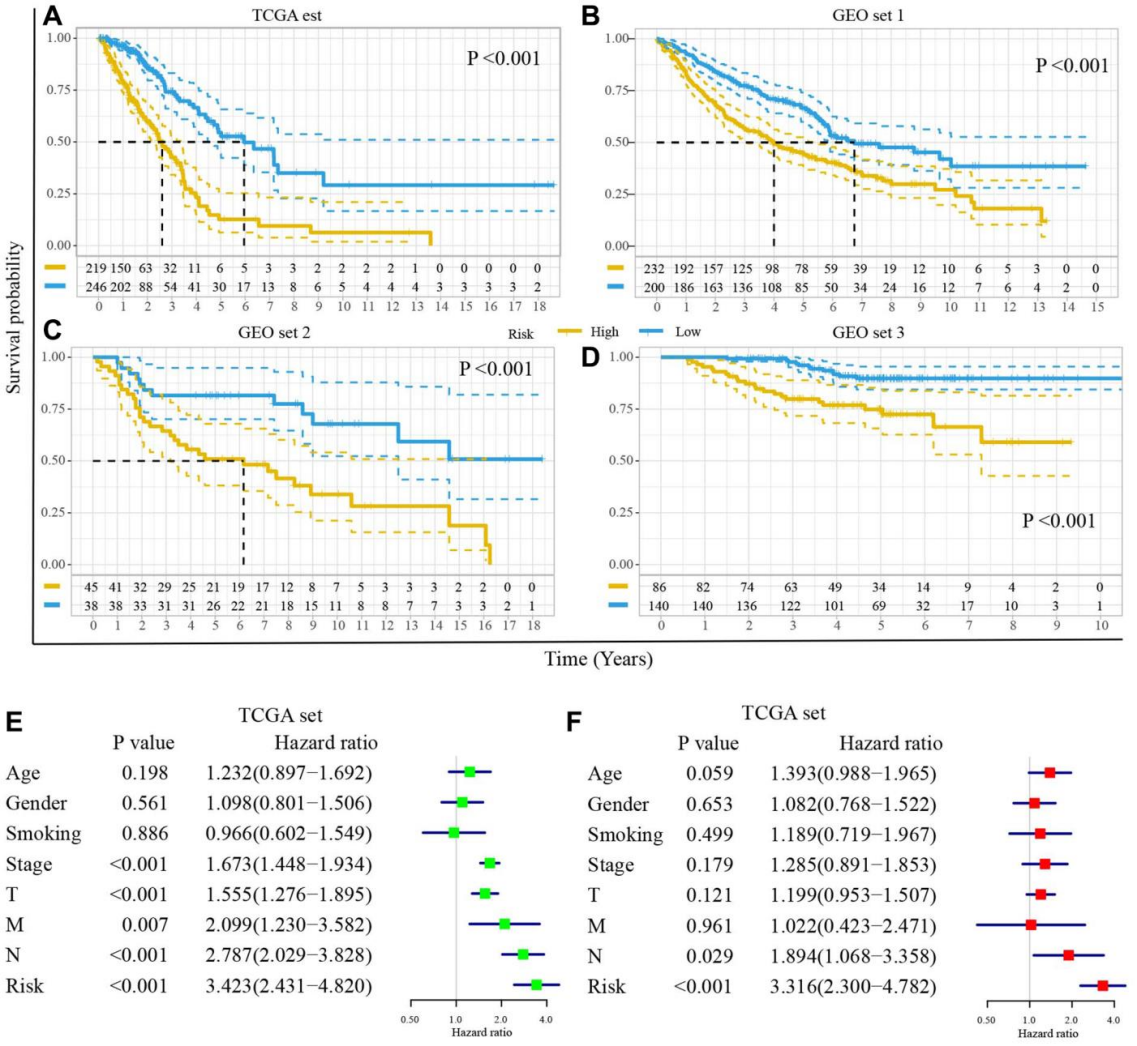
**The DRGs model is an independent prognostic factor for the prognosis of LUAD.** Survival difference between high- and low-risk groups (**A**) in TCGA set, (**B**) GEO set 1, (**C**) GEO set 2, (**D**) GEO set 3. (**E**) The univariate Cox regression analyses of risk score and clinical characteristics in TCGA set. (**F**) The multivariate Cox regression analyses of risk score and clinical characteristics in TCGA set.

The Kaplan–Meier plot in the TCGA set illustrated that the high-risk score predicted poorer clinical outcomes (*P* < 0.001, Figure 5A). It was confirmed in GEO set 1 (*P* < 0.001, Figure 5B), GEO set 2 (*P* < 0.001, Figure 5C), and GEO set 3 (*P* < 0.001, Figure 5D). Furthermore, stratification analyses demonstrated the low-risk patients had a better prognosis in each subgroup (Supplementary Figure 1).

To compare the DRGs model with clinical parameters (age, gender, stage, tumor size, lymph node metastasis, and distance metastasis), we performed the univariate and multivariate Cox regression model. The univariate Cox regression model showed that the DRGs model was an essential prognosis-related influence factor (HR = 3.423, 95% CI (2.431, 4.820), *P* < 0.001, Figure 5E). The multivariate Cox regression model demonstrated that the DRGs model was an independent prognostic factor for the OS of LUAD patients (HR = 3.316, 95% CI (2.300, 4.782), *P* < 0.001, Figure 5F). Similar results were observed in GEO set 1, 2, and 3 (Supplementary Figure 2).

### Gene set enrichment analysis (GSEA)

**Figure 6:**
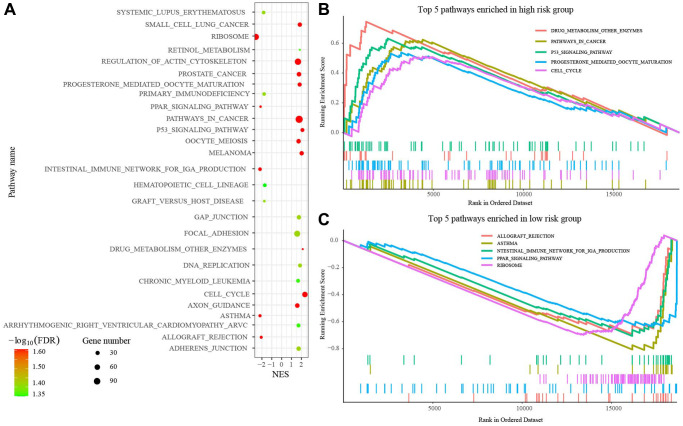
**Gene set enrichment analysis (GSEA) between high- and low-risk groups.** (**A**) 27 pathways enriched in GSEA. (**B**) Top 5 pathways enriched in high-risk group. (**C**) Top 5 pathways enriched in low-risk group. Abbreviations: FDR: False discovery rate; NES: Normalized enrichment score.

Then, we performed the GSEA to explore the altered biological roles and signaling pathways between the low- and high-risk group. A total of 27 pathways were enriched, including Pathways in cancer, P53 signaling pathway, and so on, that were highly associated with tumorigenesis and development (Figure 6A). Figure 6B, 6C presented the top 5 pathways enriched in high- and low-risk groups.

### Tumor mutational burden (TMB)

**Figure 7:**
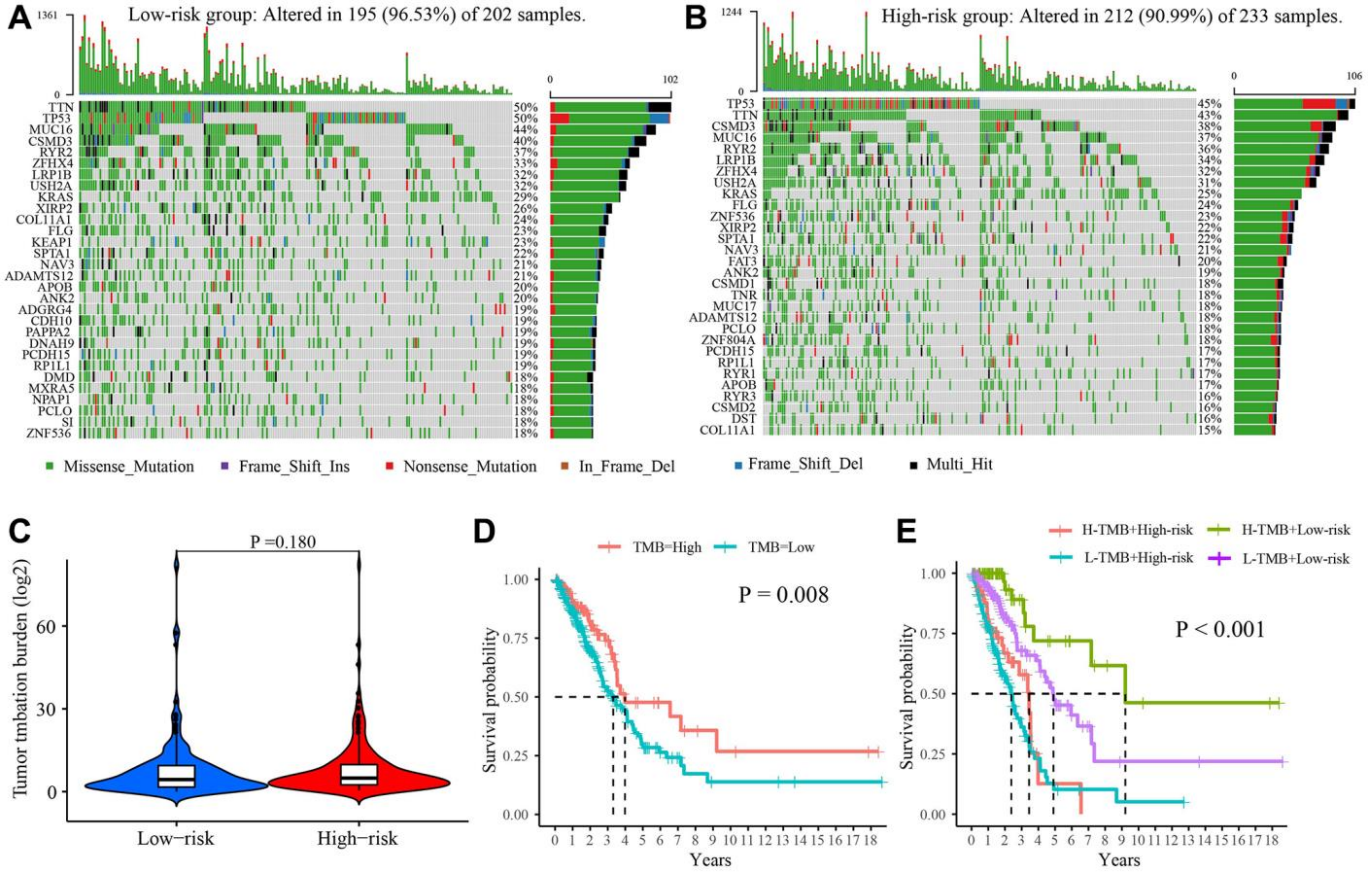
**The tumor mutation burden characteristics in low- and high-risk group.** (**A**) Mutational landscape in the low-risk group. (**B**) Mutational landscape in the high-risk group. (**C**) The difference in TMB between two groups. (**D**) Survival difference between high- and low-TMB. (**E**) Survival analysis of TMB along with risk score. Abbreviation: TMB: Tumor mutational burden.

The mutational landscape showed that the frequent mutation events in the low-risk group (96.53%) were more than that in the high-risk group (90.99%) (Figure 7A, 7B). In the high-risk group, the most frequently mutated gene was TTN (50%), followed by TP53 (50%) and MUC56 (44%). In the low-risk group, the top 3 frequently mutated genes were TP53 (45%), TTN (43%), and CSMD3 (38%). However, there was no difference in TMB between the two risk groups (*P* = 0.180, Figure 7C). Survival analysis revealed that high TMB had a better prognosis in LUAD patients (*P* = 0.008, Figure 7D). Furthermore, it could better predict prognosis when combining the TMB and the risk score (Figure 7E).

### Investigation of the relation of the DRGs model to the immune statue

**Figure 8:**
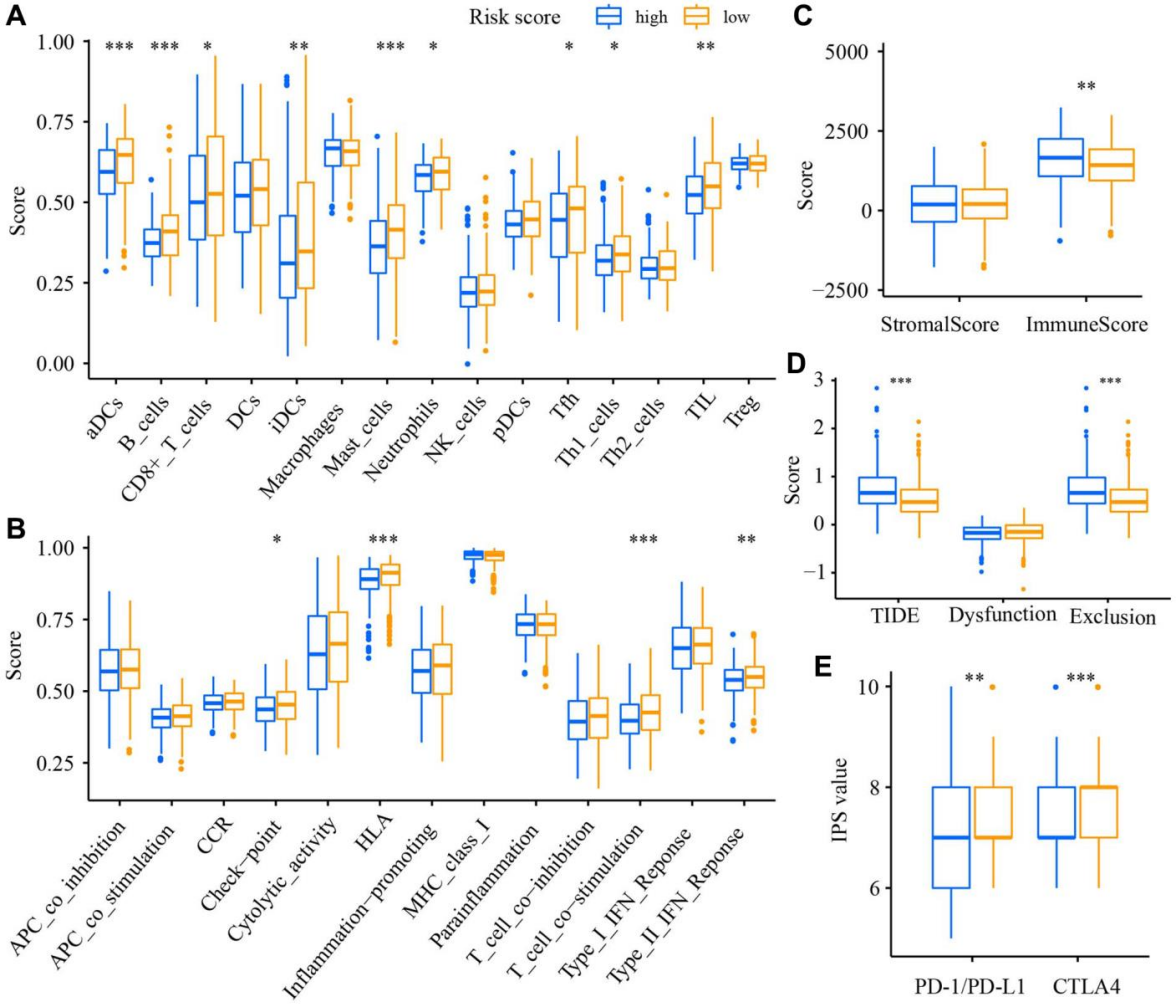
**The relation of the DRGs model to immune statue.** Comparison of the enrichment score of (**A**) TIICs, (**B**) immune-related functions, (**C**) TME, (**D**) TIDE score, (**E**) The immunophenoscore value between low- and high-risk groups. ^*^P < 0.05, ^**^P < 0.01, ^***^P < 0.001.

We applied the single-sample gene set enrichment analysis (ssGSEA) to analyze the level of 15 tumor-infiltrating immune cells (TIICs) and the activity of 13 immune-related functions. The results demonstrated that the enrichment scores of 9 TIICs (aDCs, B cells, CD8 T cells, iDCs, Mast cells, Neutrophils, Tfh, Th1 cells, TIL) in the low-risk group were significantly enhanced (Figure 8A). Similar results were observed in 4 immune-related functions (Check-point, HLA, T cell co−stimulation, Type II IFN response, Figure 8B). The immune score in the high-risk group was significantly higher than in the low-risk group (Figure 8C). The TIDE score and Exclusion score in the high-risk group were increased (Figure 8D), whereas the IPS values of PD-1/PD-L1 and CTLA4 were decreased (Figure 8E). Drug sensitivity analysis manifested a significant difference in the sensitivity of 47 anti-tumor drugs between high- and low-risk groups (Supplementary Figure 3).

### scRNA-seq analysis

**Figure 9:**
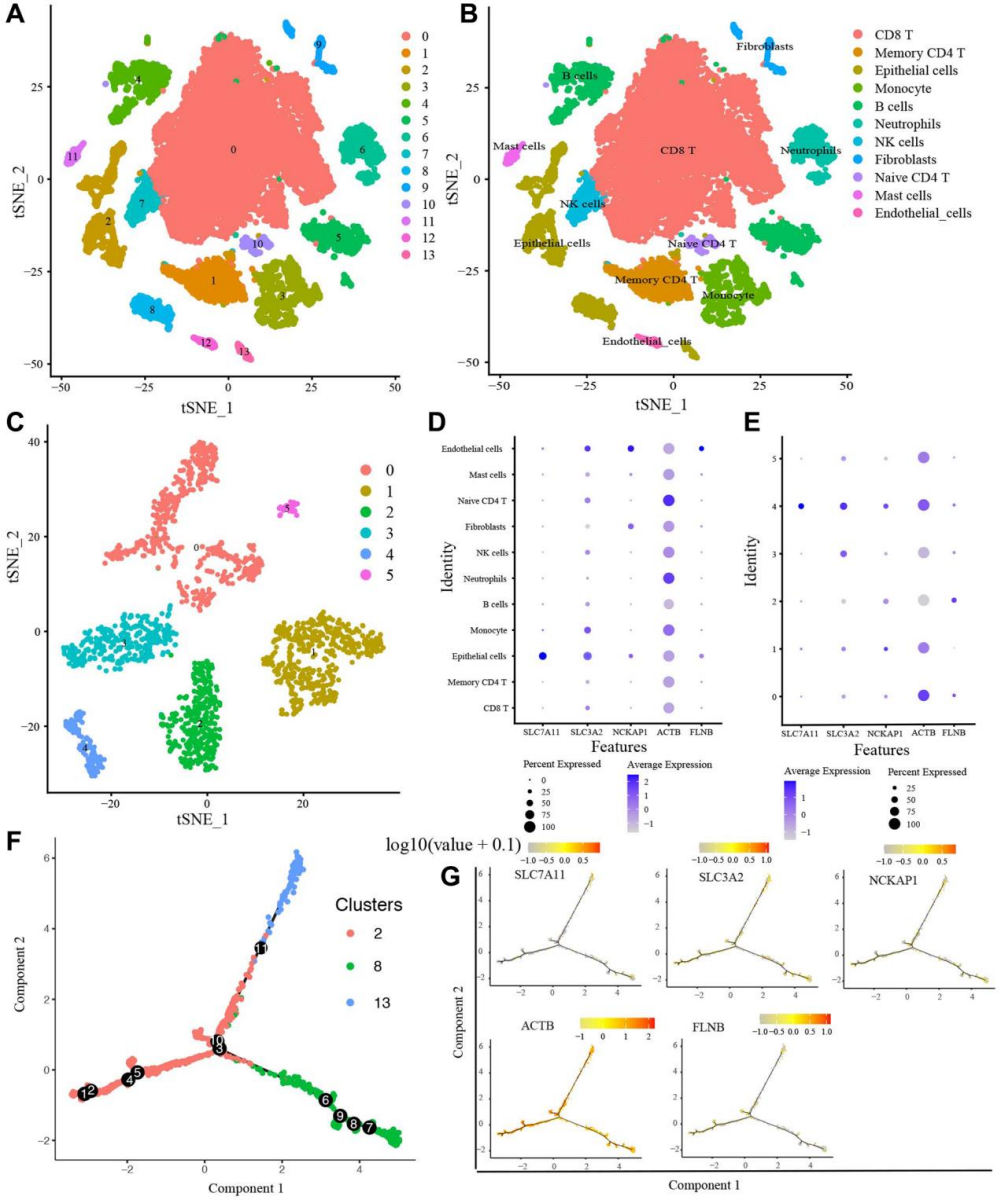
**scRNA-seq data analysis.** (**A**) The t-SNE algorithm divided the cells into 14 clusters by 20 principal components. (**B**) The tSNE plot revealing 14 clusters was annotated into 11 different cell types. (**C**) The t-SNE algorithm divided Epithelial cells into 6 sub-clusters. (**D**) The expression of 5 candidate DRGs in 11 cell types, (**E**) in 6 Epithelial cells sub-clusters. (**F**) The trajectory analysis of Epithelial cells with top 10 marker genes. (**G**) 5 candidate DRGs expression in different developmental states. Abbreviations: ScRNA-seq: Single-cell RNA sequencing; t-SNE: t-Stochastic Neighbor Embedding.

Before filtering, there were 45,632 features for 23,747 cells in the 8 LUAD samples. Then, we performed data standardization and quality control and finally selected 18,977 cells and the top 2000 highly expressed and variable genes for further analysis. The PCA for reducing data dimensionality reduction revealed no noticeable separation trend of cells. Nonlinear dimension reduction was performed with the t-Stochastic Neighbor Embedding (t-SNE) algorithm, which successfully clustered the cells into 14 clusters (Figure 9A). Then, we annotated all clusters, and 11 cell types were identified (Figure 9B). In addition, the expression level of SLC7A11 and SLC3A2 were most abundant in Epithelial cells (Figure 9D). NCKAP1 and FLNB had the highest expression in Endothelial cells. ACTB was expressed in most cell types. However, the expression level in Naive CD4 T was the richest. In this study, cluster 2, 8, and 13 were annotated into Epithelial cells, which mainly contained cancer cells and cancer stem cells. Thence, we included the Epithelial cells in the pseudo-time cell differentiation trajectory analysis. The results were presented in Figure 9F and demonstrated the evolutionary pattern of Epithelial cells. Based on the ordering of pseudotime, Epithelial cells (cluster 2) were divided into Epithelial cells (cluster 8) and Epithelial cells (cluster 13). Figure 9G showed the 5 candidate DRGs expression in different developmental states. Finally, we further analyzed the Epithelial cells with the t-SNE algorithm and clustered Epithelial cells into 6 sub-clusters (Figure 9C). The expression of SLC7A11, SLC3A2, NCKAP1, and ACTB in sub-cluster 4 was highest, whereas FLNB in sub-cluster 2 was highest (Figure 9E).

### Construction of a prognostic nomogram

**Figure 10:**
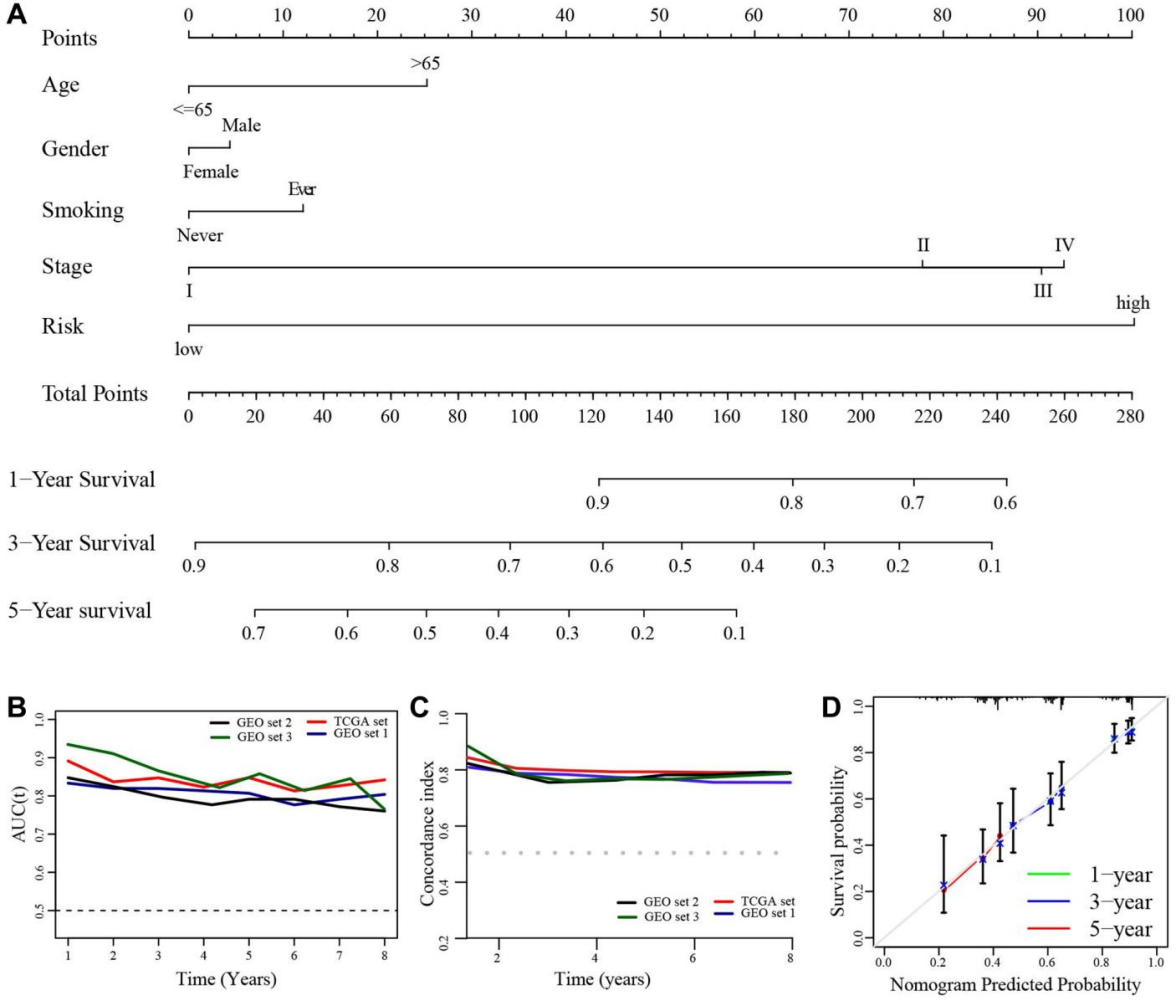
**Construction of a prognostic nomogram.** (**A**) A nomogram for predicting the 1-, 3-, and 5-year overall survival rate in LUAD patients. (**B**) The time-dependent AUC value. (**C**) The time-dependent C-index. (**D**) The calibration plot.

Using clinical characteristics (age, gender, smoking, TNM stage) and risk score, we develop a prognostic nomogram in TCGA set for predicting 1-, 3-, and 5-year OS of LUAD patients (Figure 10A). The AUCs and c-index in each set were more than 0.7 (Figure 10B, 10C). In addition, the calibration plot showed an optimally fit with the ideal model (Figure 10D).

## DISCUSSION

It has been reported that the incidence rate of LC is continuously rising because of smoking, air pollution, and other factors. Also, the morbidity of LUAD, the most common pathological subtype of LC, is increasing [1, 10]. Despite emerging advanced diagnostic and therapeutic means which have extensively upgraded the long-term quality of life and survival rate of LUAD patients, the 5-year survival rate is still <20% [4, 5]. The most commonly used method for clinical treatment and prognosis prediction in LUAD is the TNM stage. Nevertheless, numerous studies have shown that, although patients had the same TNM stage and therapy strategy, the prognosis was different, indicating that prognosis prediction only relying on the TNM stage maybe not be enough to evaluate the clinical outcomes in LC [11]. Therefore, identifying more accurate tumor-specific biomarkers of LUAD for further guiding clinical decisions and prognostic evaluation remains a top priority.

Recently, a novel cell death model: disulfidptosis, has been proposed, which was induced by disulfide stress and markedly distinguished from any of the commonly studied forms of regulated cell death [8, 9]. In the cell death model, the excessive accumulation of intracellular disulfide molecules causes disulfide stress and bond to actin cytoskeleton proteins, subsequently leading to actin network collapse and cell death. In the process, NADPH could suppress disulfidptosis via resolving disulfide bonding to actin cytoskeleton proteins. There is no doubt that the concept would benefit a further and more comprehensive understanding of cell death mechanisms, which may provide a practicable therapeutic means by targeting disulfidptosis in cancer treatment. However, up to now, no research has been reported on the role of disulfidptosis in LUAD. Herein, we would investigate the expression of DRGs in LUAD and the predictive value of DRGs in LUAD.

Firstly, we analyzed the differential expression of DRGs between normal and LUAD tissues and found the expression of 22 DGRs except for ACTN4 in LUAD tissues was significantly increased/decreased compared with normal tissues, implying the process of disulfidptosis in LUAD underwent drastically changed. Then, we took advantage of LASSO analysis and multivariate Cox regression analysis to develop a DRGs model for predicting the prognosis of LUAD patients with 5 candidate DRGs (ACTB, FLNB, NCKAP1, SLC3A2, SLC7A11). The AUC value and C-index based on the DRGs model in 4 sets were all more than 0.7 and significantly superior to that based on clinical parameters (age, gender, TNM stage, and smoking). Furthermore, the calibration plot demonstrated the consistency between the survival rate predicted by the DRGs model and the actual survival rate. All data suggested that the established prognostic model is stable enough to estimate the prognosis of LUAD patients.

We divided all samples into low- and high-risk groups according to the risk-score formula. The survival analysis illustrated that patients in the high-risk group exerted poorer clinical outcomes. And stratification analyses confirmed the results. Moreover, the univariate and multivariate Cox regression model indicated that the risk score was an independent prognostic factor in LUAD patients. The above data manifested that disulfidptosis was highly associated with the prognosis of LUAD patients, and the DRGs model could provide a good prediction of the clinical outcomes.

To determine the potential biological process changes between the low- and high-risk groups, we executed a GSEA analysis. The results showed 27 pathways were enriched. Of note, several immune-related pathways were identified in the low-risk group, including Primary immunodeficiency, Intestinal immune network for IgA production, etc. For further investigating the relation of the DRGs model to the immune status, we appraised the difference in the level of TIICs, the activity of immune-related functions, and the response to immunotherapy between the two groups. Interestingly, in the low-risk group, patients had a higher level of TIICs and better immune function, which may be why those patients had better treatment responses to immune therapy and better prognosis.

The application of Next Generation Sequencing (NGS) provides a practicable means for exploring human cell transcription patterns [12]. Traditional method is cost-effective and measures the total RNA of all cells. Still, it fails to consider the heterogeneity of cancer cells, which poses substantial challenges in diagnosing and treating cancer [13]. To uncover the expression profiles of DRGs in different cell types in LUAD tissue, we performed scRNA-seq analysis. All cells were clustered into 14 clusters and annotated to 11 cell types. CD8 T cell was the most abundant cell. CD8 T cells are classified into naive like, cytotoxic, and dysfunctional CD8 T cells based on their differentiation status. CD8 T cells have the ability to selectively detect and eradicate cancer cells. However, the sustained antigen stimulation of the tumor itself is considered one of the main driving factors for T cell dysfunction. In order to eliminate the tumor cells, T cells will enter the tumor microenvironment from the peripheral circulation. Because of this, the proportion of CD8 T cells in the tumor microenvironment will significantly increase. In addition, three clusters (2, 8, 13) were annotated to Epithelial cells. Tumor Epithelial cells mainly contain cancer cells and cancer stem cells. Then, we pictured the transcriptional differentiation trajectory of the epithelial cells. The results demonstrated that Epithelial cells (clusters 2) differentiate into two clusters (cluster 8 and 13), implying the heterogeneity of cancer cells.

Herein, we developed a DRGs model to predict the OS in LUAD patients. However, some limitations still need to be addressed. (1) All data were gathered from two public databases: TCGA and GEO, and lacking sufficient experimental evidence and animal models to verify the results, which may lead to potential selection bias. (2) This study was retrospective, and prospective validations were still needed. (3) All data used in the study were from microarray expression and RNA-seq (RNA sequencing). And the specific role of disulfidptosis progress remains relatively enigmatic and warrants further investigation. (4) Herein, we performed stratification analyses and determined the significant survival difference between the low- and high-risk groups. However, due to lacking information of therapies like surgery, targeted therapy, and immunotherapy in most patients, we could not homogenize the treatment and evaluate the predictive effectiveness and accuracy of the IRGPs signature in patients with surgery, targeted therapy, and immunotherapy. It may bring biased prognosis predictions.

Together, in the study, we investigated the expression profile of DRGs and constructed a predictive DRGs model for predicting the prognosis of LUAD patients. Evidence showed that the model was stable and reliable for predicting LUAD prognosis. Our research provided a novel understanding of the predictive value of disulfidptosis progress in LUAD and will be helpful for the prognosis evaluation of LUAD patients.

## MATERIALS AND METHODS

### Patients set

Four independent sets, including 1306 samples, were collected from TCGA and GEO databases in the study. TCGA set (*n* = 465) was from TCGA-LUAD and level 3 RNA sequencing. GEO set 1 (*n* = 432), GEO set 2 (*n* = 83), and GEO set 3 (*n* = 226) were from GSE68465 [14], GSE30219 [15], and GSE31210 [16], respectively. The gene expression profile in GSE68465, GSE30219, GSE31210 was raw data and normalized with the robust multi-array average algorithm via the R package Affy (3.17). The corresponding clinical data of all patients were also collected. Patients lacking pathologic diagnosis, survival time, or survival status would be removed. All patients’ detailed demographic and baseline information was presented in Table 1. ScRNA-seq data from 8 human LUAD samples were gathered from the GSE171145 [17] dataset in the GEO database. Disulfidptosis-related genes were obtained from previous literature [8, 9, 18, 19].

**Table 1:** The baseline characteristics of lung adenocarcinoma patients in this study.

**Parameter**	**TCGA cohort**	**GEO cohort 1**	**GEO cohort 2**	**GEO cohort 3**
Database	TCGA-LUAD	GSE68465	GSE30219	GSE31210
Age				
≤65	235 (50.53%)	224 (51.85%)	60 (72.29%)	176 (77.88%)
>65	230 (49.46%)	208 (48.15%)	23 (27.71%)	50 (22.12%)
Gender				
Female	248 (53.33%)	214 (49.54%)	36 (43.37%)	121 (53.54%)
Male	217 (46.67%)	218 (50.46%)	47 (56.63%)	105 (46.46%)
Smoking				
Never	62 (13.33%)	50 (11.57%)	0	115 (55.88%)
Ever	384 (82.58%)	290 (67.13%)	0	111 (49.12%)
NA	19 (4.08%)	92 (21.30%)	83 (100%)	0
TNM stage				
I	264 (56.77%)	149 (34.50%)	64 (77.11%)	114 (50.44%)
II	103 (22.15%)	216 (50.00%)	13 (15.66%)	78 (34.51%)
III	73 (15.70%)	67 (15.50%)	6 (7.23%)	34 (15.05%)
IV	25 (5.38%)	0	0	0
Tumor size				
T1	162 (34.84%)	148 (34.26%)	56 (67.47%)	NA
T2	241 (51.83%)	245 (56.71%)	17 (20.48%)	NA
T3	44 (9.46%)	28 (6.48%)	10 (12.05%)	NA
T4	18 (3.87%)	11 (2.55%)	0	NA
NA	0	0	0	226 (100%)
Lymph node				
N0	316 (67.96%)	298 (68.98%)	73 (87.95%)	NA
N1-3	149 (32.04%)	134 (31.02%)	10 (12.05%)	NA
NA	0	0	0	226 (100%)
Metastasis				
M0	442 (95.05%)	432 (100%)	83 (100%)	NA
M1	23 (4.95%)	0	0	NA
NA	0	0	0	226 (100%)
Survival status				
Alive	305 (65.60%)	203 (46.99%)	40 (48.19%)	191 (84.51%)
Dead	160 (34.40%)	229 (53.01%)	43 (51.81%)	35 (15.49%)
Risk score				
Low	219 (47.10%)	232 (53.70%)	45 (54.22%)	86 (38.05%)
High	246 (52.90%)	200 (46.30%)	38 (45.78%)	140 (61.95%)
Total	465 (100%)	432 (100%)	83 (100%)	226 (100%)

Abbreviations: TCGA: The Cancer Genome Atlas; GEO: Gene Expression Omnibus; NA: represents information not available.

### Establishment and evaluation of a disulfidptosis-related genes model (DRGs) model

In the TCGA set, we investigated the difference in the expression of 23 DRGs between normal and LUAD tissues with the Wilcoxon rank-sum test. Then, we preliminary identified prognosis-related genes among 23 DRGs using the least absolute shrinkage and selection operator (LASSO) regression analysis with R package “glmnet” (4.0.2). Under the minimum lambda, genes with coefficient ≠ 0 were chosen for screening genes. The iteration was set as 1,000 and 10-fold cross-validations to avoid overfitting. Then, the screened genes were subjected to the multivariate Cox regression analysis to filter candidate genes and develop a DRGs model for predicting the prognosis of LUAD. The risk score was calculated as Risk score = h0(t) × exp (β1X1 + β2X2 + … + βnXn), where β refers to the regression coefficient; X represented the gene expression level; h0(t) is the benchmark risk function. Also, a risk score formula was formed, and patients’ risk scores were calculated. Finally, we used the “surv cutpoint” functions of the package “Survminer” (0.4.9) in R to determine the optimal cut-off value of risk score, which divided patients into low- and high-risk groups. Based on the cut-off value, patients in GEO set 1, 2, and 3 were also divided into two groups.

The AUC value and the c-index in four sets were calculated to assess the predictive capacity of the DRGs model. The calibration plot with a boot-strapping set of 1,000 resamples was also pictured to investigate the predictive accuracy of the DRGs model.

### GSEA

The GSEA was performed to explore the altered biological roles and signaling pathways between the low- and high-risk group with the R package “clusterProfiler” (4.2.2). The “c2.cp.v7.2.symbols.gmt (Curated)” was selected as annotated gene set. The false discovery rate (FDR) <0.05 was set as the threshold.

### Assessment of the relation of the DRGs model to the immune statute and drug sensitivity

The R package: GSVA (3.1.7) was applied to carry out the ssGSEA reflecting the level of TIICs and the activity of immune-related functions. The R package: “ESTIMATE” (1.0.13) was used to calculate the immune and stromal score in the tumor microenvironment. The immunophenoscore (IPS) value of samples was gathered from The Cancer Immunome Atlas (TCIA, https://tcia.at) to present the response to immune checkpoint inhibitors treatment [20]. The TIDE score was collected from Tumor Immune Dysfunction and Exclusion (TIDE, http://tide.dfci.harvard.edu). The higher TIDE score represents the worse response to immune therapy.

We took advantage of the R package “pRRophetic” (version 6) [21] to calculate the semi-inhibitory concentration values of commonly used anti-tumor drugs for LUAD to look for variations in the efficacy of medicines between the two groups. *P* < 0.01 was set as the cut-off value.

### TMB

The DNA somatic mutation data of corresponding LUAD patients were also downloaded from TCGA and further analyzed with the “maftools” R package (3.17). The ChromPlot function in the “maftools” R package was used to visualize the output results. The results were presented with waterfall diagram to show the variation distribution of genes with high somatic mutation frequency in LUAD samples and two different groups. Further, it was used to evaluate the difference in survival of the patients classified in the high-TMB and low-TMB groups.

### scRNA-seq data processing

The R package: “Seurat” (3.4) [22] was used to analyze the transcript count matrix for quality control and preliminary data exploration. The filtering threshold was set as follows:

Excluding genes detected in less than 3 cells
Excluding cells with <50 genes detected
Excluding cells with >10% mitochondrial gene expression

Then, the expression profiles were normalized with the Log Normalization algorithm and were subsequently normalized using a linear regression model. The top 2000 highly expressed and variable genes were selected for PCA to determine significant and influential dimensions. The t-SNE algorithm was used to reduce the dimension of the top 20 principal components and gather major cell clusters. The marker genes between difference clusters were identified with |log2 (fold change) |>1 and adjusted *P* value < 0.05 as the threshold. Cell annotation was carried out with the “SingleR” package (3.17) [23] and reports from the literature [24–26]. Finally, single-cell trajectory analysis was performed with the “Monocle 2 algorithm” [27].

### Statistical analysis

The categorical data were presented as Numbers and compared with the chi-square test. The measurement data were presented as Mean ± standard deviation (SD) and compared with the Wilcoxon rank-sum test. Correlation analysis was performed with the Spearman correlation test. Survival analysis was performed with the Kaplan–Meier plot and compared by the log-rank method. Finally, the univariate and multivariate Cox regression analysis determined the independent prognostic predictors. *P* < 0.05 was set as the cut-off value. All statistical analyses were conducted with R 4.1.1 (https://www.r-project.org).

### Availability of data and materials

Bulk RNA-seq data were analyzed in this study, this data can be found at: The Cancer Genome Atlas (TCGA-LUAD, https://portal.gdc.cancer.gov) and Gene Expression Omnibus (GSE68465, GSE31210, and GSE 30219; https://www.ncbi.nlm.nih.gov). ScRNA-seq data were collected from GEO-GSE171145.

## Supplementary Materials

Supplementary Figures
