# Supplementary material for: The expression and prognostic value of disulfidptosis progress in lung adenocarcinoma
Source: Aging (Albany NY). 2023 Aug 7;15(15):7741–59. doi: 10.18632/aging.204938 (PMC10457049; doi:10.18632/aging.204938)
Supplement: Supplementary Figures [file aging-15-204938-s001.pdf]

## SUPPLEMENTARY FIGURES

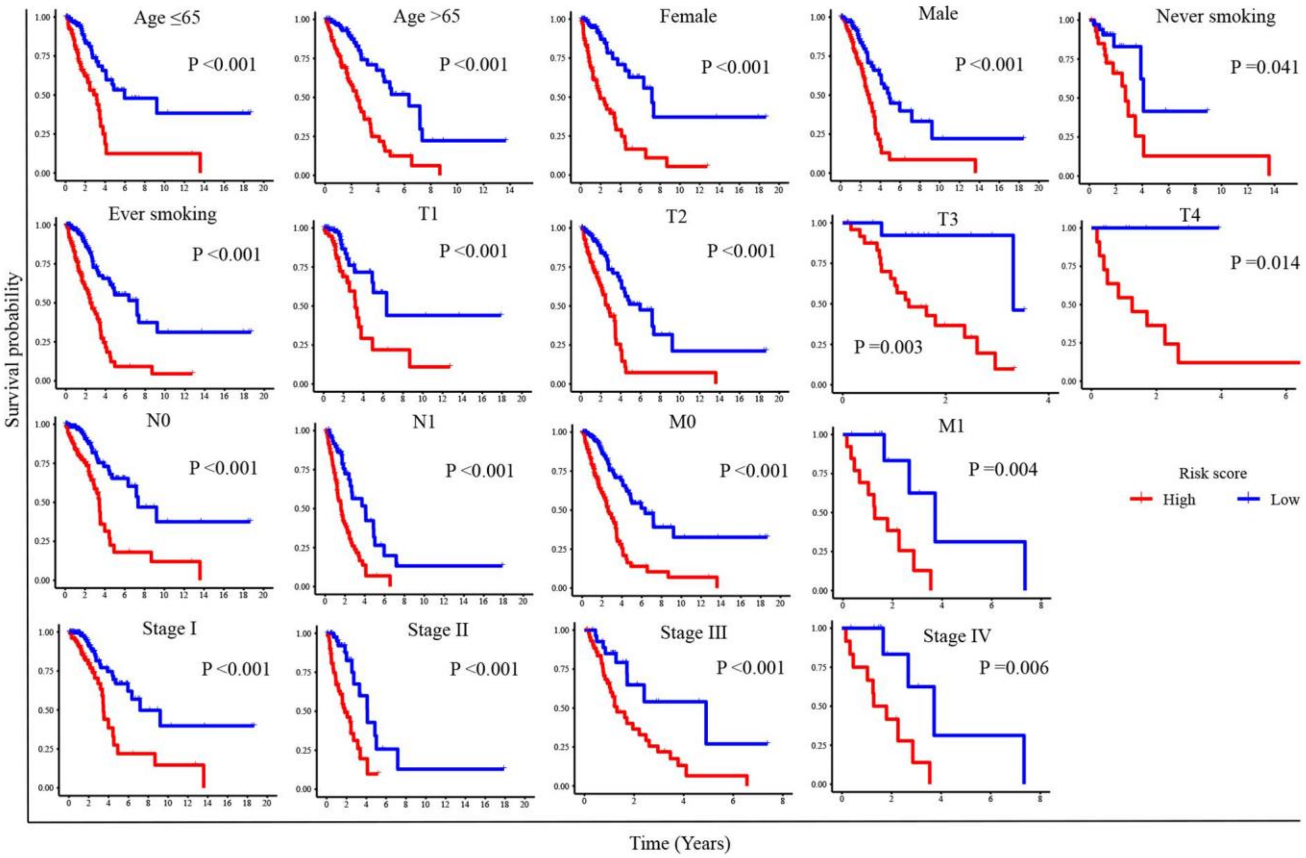


**Supplementary Figure 1. Stratification analyses of overall survival between high- and low-risk patients in different subgroup.**

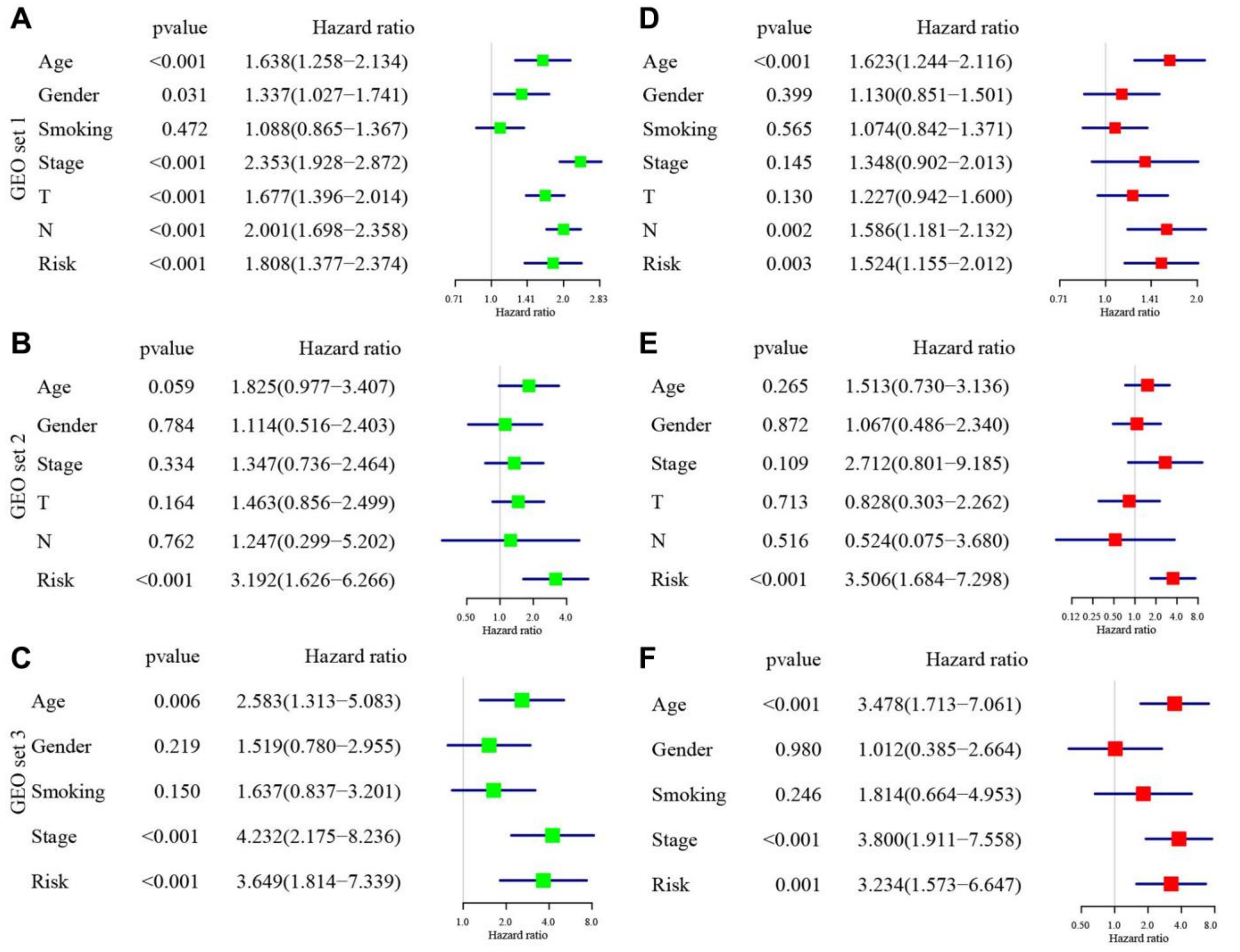


**Supplementary Figure 2.** The univariate Cox regression analysis of risk score and clinical characteristics in (**A**) GEO set 1, (**B**) GEO set 2, (**C**) GEO set 3. The multivariate Cox regression analysis of risk score and clinical characteristics in (**D**) GEO set 1, (**E**) GEO set 2, (**F**) GEO set 3.

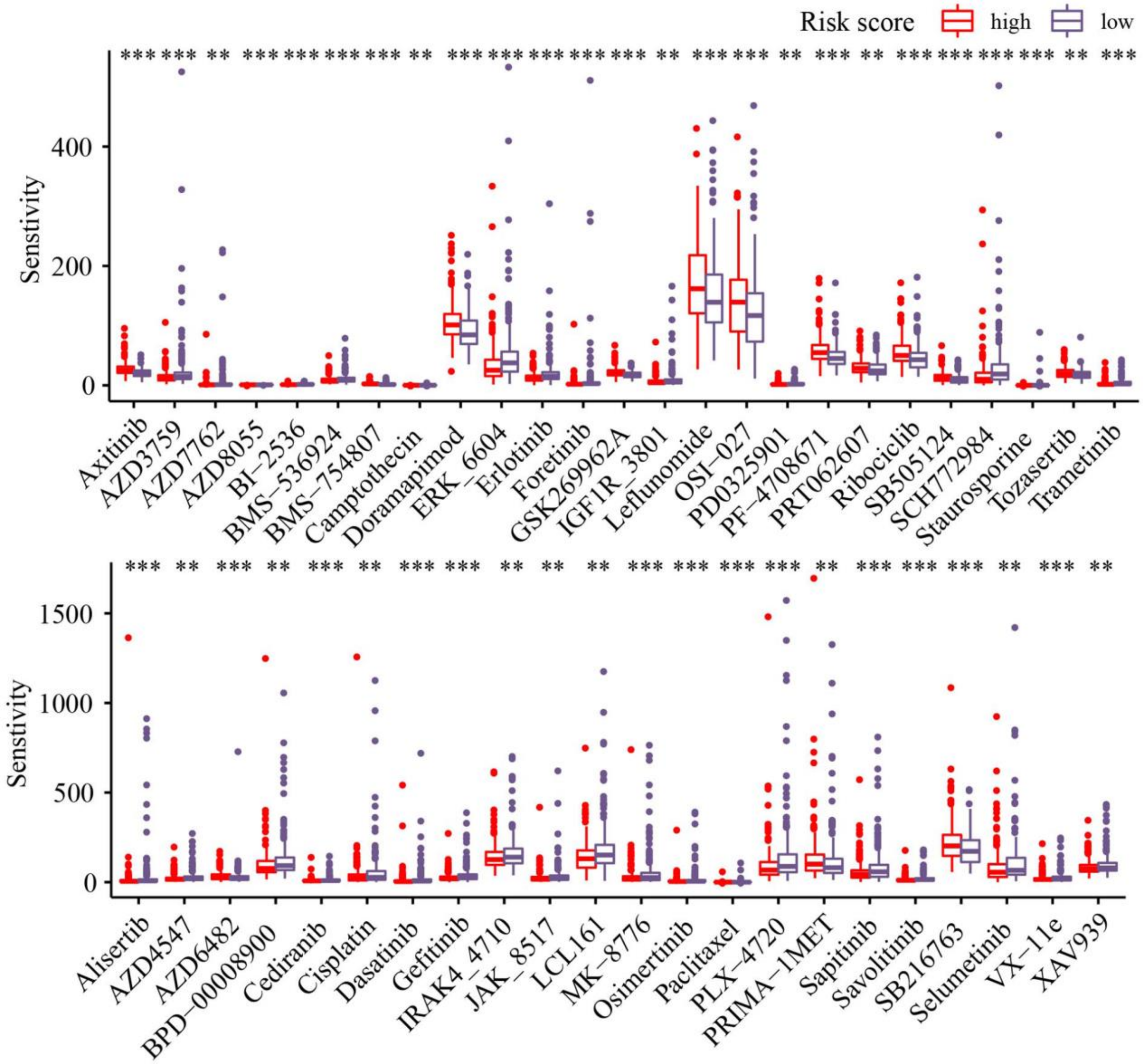


**Supplementary Figure 3. Drug sensitivity analysis between high- and low-risk patients.**
